# Miscellany

**Published:** 1905-10

**Authors:** 


### Miscellany.

An Automatic Anæsthetic Capsule-Holder and Breaker (designed by Mr. Vernon Knowles).—There is little doubt but that the capsule form of the more volatile anæsthetics, such as ethyl chloride, somnoform, etc., has "come to stay," since the advantages obtained are many and obvious.

In the first place, they can be kept any length of time, in any climate, without the slightest risk of deterioration, or diminution of their contents. Then, again, exact and known quantities can be relied upon, a very important factor, as the risk of overdosage, with its distressing and sometimes fatal symptoms, is entirely eliminated. They are open to one great objection,—viz., the difficulty of their manipulation.

Some makers advise cutting off the tips with a pair of scissors; others, that the necks should be nicked with a file. Neither are satisfactory. Not only is there danger of the operator being wounded by glass splinters, but ofttimes their contents are lost through fracture of the capsule, or by being forcibly expelled.

To overcome this, various inhalers have been introduced containing contrivances whereby the capsules can be fractured inside the same. This is an objectionable, if not a dangerous method, in that particles of glass are scattered in all directions. This device overcomes these difficulties in a simple and practical manner.

It is small and compact, and, being of metal throughout, is unbreakable; owing to its simplicity of construction, it is little liable to get out of order.

It consists of four parts, which when put together form a metal case completely enclosing the capsule,—the body which holds the capsule, a sheath which keeps it in position, a ball-and-socket nozzle, and a nozzle cap. These are held together by bayonet catches.

When the capsule is inserted and the parts are put together, the neck of the capsule projects into the ball-and-socket nozzle; it therefore follows that when the latter is deflected in any direction the neck is fractured, and the contents projected on to or into any medium that may be selected for presenting the anæsthetic, a properly placed fine gauze screen effectively holding back any glass particles. The device is manufactured by the Dental Manufacturing Company, of London.—(*Dental Record*, vol. xxv., July, 1905, p. 342.)

---

THE INFLUENCE OF THE FIRST AND SECOND DENTITION PERIODS IN THE ETIOLOGY OF EPILEPSY.—In the course of an article bearing this title in the *Medical News* of December 10, 1904, Spratling gives us the following views which bear on treatment. He thinks it is always a mistake to regard the convulsions of dentition, or the convulsions due to any other cause in early life, in any other than a serious light. They are never positively benign—at least, we have no right to regard them so. That infants who have convulsions escape serious consequences in the future is always a matter for congratulation, but the physician should never assume that this is the outcome to be expected. When disease tendencies are so strongly marked as these morbid manifestations so plainly indicate, the most constant care and treatment should be undertaken at once in every case with a view to preventing epilepsy, or idiocy, or insanity, or other states of degeneracy destined to destroy the mental life of the individual in question.

In conclusion, the author's views in the matter may be briefly summarized as follows:

1. Difficult dentition—*i.e.*, the piercing of the gums by the tooth

—may, in suitable subjects, constitute a sufficient irritant to cause convulsions.

2. In suitable subjects these convulsions may ultimately lead to epilepsy.

3. By suitable subjects is meant infants who inherit a neuropathic tendency to disease; whose parents have epilepsy, or insanity, or who are alcoholic, or suffer from some other general vice that could be transmitted to the offspring in some form capable of vitiating its powers of resistance to disease.

4. The author does not believe that difficult dentition alone in a child who inherited no ancestral taints, and who at its birth is free from a tendency to nervous disease, can cause epilepsy.

5. Great caution must always be exerted to lay the true cause in cases of this kind where it belongs; for the reason that gastro-intestinal disorders, the sequelæ of the eruptive fevers, and other factors common at this age, may produce similar results.

---

A Novel Head-Rest, especially useful in Tooth-Extraction.—An article in the June number, 1905, of C. Ash & Sons' Quarterly Circular, page 189, credited to Mr. W. Orr Gray, L.D.S. (Eng.), D.D.S., describes and illustrates a novel head-rest, invented by Dr. Frank Nyulasy, of Melbourne, Australia, especially useful in tooth-extraction under a general anæsthetic. This head-rest is shaped very much like a bowl, and is intended to be fixed, by means of adjustable arms, to an ordinary surgical operating-table, or to a common deal table, to support the patient's head while he is recumbent upon the table. The bowl-like rest is fixed a few inches below the level of the table, and supports the head comfortably and firmly in a position not only favorable to operations within the mouth, but in a position especially favorable to easy respiration. It was originally designed to facilitate operations for post-nasal growths, for operations upon the roof of the mouth and upper jaw, for excision of the larynx, and for endoscopic examinations of the œsophagus. Its chief advantages to the dentist are: (1) The absolute prevention of all danger from ingress of teeth, roots, etc., to the respiratory passages. (2) Complete and unhampered use of the respiratory mechanism, in contradistinction to the strained position of the anterior muscles of the neck when the patient is in a dental chair. (3) The facility with which lower back roots or impacted wisdom-teeth can be removed, as well as all the other teeth. (4) The almost total prevention of the passage of blood to the stomach during anæsthesia.

The recumbent position is especially favorable to safety in administration of general anæsthetics, and to rapid recovery from their effects.

The article closes with the remark that "for further information as to the head-rest, the *British Medical Journal* of April 20, 1904, may be consulted."

